# Hematological Parameters, Lipid Profile, and Cardiovascular Risk Analysis Among Genotype-Controlled Indigenous Kiwcha Men and Women Living at Low and High Altitudes

**DOI:** 10.3389/fphys.2021.749006

**Published:** 2021-10-25

**Authors:** Esteban Ortiz-Prado, David Portilla, Johanna Mosquera-Moscoso, Katherine Simbaña-Rivera, Diego Duta, Israel Ochoa, German Burgos, Juan S. Izquierdo-Condoy, Eduardo Vásconez, Manuel Calvopiña, Ginés Viscor

**Affiliations:** ^1^One Health Research Group, Faculty of Medicine, Universidad de las Americas, Quito, Ecuador; ^2^Department of Cell Biology, Physiology and Immunology, Universidad de Barcelona, Barcelona, Spain; ^3^General Ward, Limoncocha Community Health Unit, Limoncocha, Ecuador; ^4^General Ward, Oyacachi Community Health Unit, Oyacachi, Ecuador; ^5^Faculty of Medicine, Universidad de Las Americas, Quito, Ecuador

**Keywords:** high altitude, hypoxia, hematological profile, adaptation, lipid profile, cardiovascular risk

## Abstract

**Introduction:** Human adaptation to high altitude is due to characteristic adjustments at every physiological level. Differences in lipid profile and cardiovascular risk factors in altitude dwellers have been previously explored. Nevertheless, there are no reports available on genotype-controlled matches among different altitude-adapted indigenous populations.

**Objective:** To explore the possible differences in plasma lipid profile and cardiovascular risk among autochthonous Kiwcha people inhabitants of low and high-altitude locations.

**Methodology:** A cross-sectional analysis of plasmatic lipid profiles and cardiovascular risk factors in lowland Kiwchas from Limoncocha (230 m) and high-altitude Kiwchas from Oyacachi (3,800 m).

**Results:** In the low altitude group, 66% were women (*n* = 78) and 34% (*n* = 40) were men, whereas in the high altitude group, 59% (*n* = 56) were women and 41% (*n* = 41%) were men. We found the proportion of overweight and obese individuals to be higher among low altitude dwellers (*p* < 0.05). Red blood cells (RBCs), hemoglobin concentration, and SpO_2_% were higher among high altitude dwellers and the erythrocyte size was found to be smaller at high altitude. The group located at low altitude also showed lower levels of plasma cholesterol, low-density lipoprotein (LDL), and high-density lipoprotein (HDL), but most of these differences are not influenced by gender or elevation.

**Conclusions:** Living at an altitude elicits well-known adaptive physiological changes such as erythrocyte count, hemoglobin concentration, hematocrit level, and serum glucose level. We also report clinical differences in the plasma lipid profile, with higher levels of cholesterol, HDL, and LDL in inhabitants of the Andes Mountain vs. their Amazonian basin peers. Despite this, we did not find significant differences in cardiovascular risk.

## Introduction

Humans have developed adaptive mechanisms that allow them to live under extreme conditions. These conditions include cold and harsh environments such as those found at high-altitude locations. It has been difficult to define at which elevation the effects of high-altitude become more severe and where the threshold is located in terms of mild or severe hypoxia (West et al., 2007). Imray et al. (2011) used classification of high-altitude exposure in accordance with recommendations from the International Society of Mountain Medicine, a categorization that seems to be the most pragmatic (Imray et al., 2011). The author defined low altitude as everything that is located below 1,500 m, moderate or intermediate altitude from 1,500 to 2,500 m, high-altitude from 2,500 to 3,500 m, very high-altitude from 3,500 to 5,800 m, extreme high-altitude above 5,800 m, and death zone above 8,000 m (Imray et al., 2011).

Worldwide, more than 140 million people reside above 2,500 m (Pasha and Newman, 2010). Studying high-altitude dwellers is essential to understand the environmental, physiological, and genetic factors that are linked to the incidence and prevalence of different maladies in these populations (Miranda et al., 2019).

Acute and chronic exposure to high altitude has a variety of effects on human physiology and can be the cause of the occurrence of many diseases (Milledge, 2020). Barometric pressure decreases exponentially with increasing altitude. Consequently, the partial pressure of oxygen also decreases, despite which the composition of gases in the atmosphere remains unaltered. The physiological consequences of this reduction in oxygen availability begin to be noticeable, even at rest, from an altitude of 2,500 m (Ortiz-Prado et al., 2019). For that reason, residents at high altitudes have physiological and morphological adaptations that allow them to deal with these environmental conditions, whereas habitual residents at low altitudes must acclimatize once they ascend to these elevations (West, 2006). The anatomical, ventilatory, and cardiovascular differences between populations (residents at low vs. high altitudes) have been widely described; nevertheless, it is still unclear if those physiological alterations act as protective (“strain”) or risk (“stress”) factors (Sherpa et al., 2011; Ortiz-Prado et al., 2017; Dhiman et al., 2018).

One of the most controversial issues is the potentially higher cardiovascular risk among high altitude dwellers. The cardiovascular health of populations permanently living at high altitude may not only depend on the degree of altitude adaptation reached by this particular population but also on lifestyle factors and genetic predisposition (Aryal et al., 2015). In particular, various risk factors can be noticed among Andean highland populations including excessive erythrocytosis (Monge's disease or chronic mountain sickness) and a hypercoagulable-prothrombotic state linked to a higher incidence of thrombosis, probably due to venous blood flow stasis and secondary polycythemia (Zangari et al., 2013). On the other hand, factors such as hypercholesterolemia and hyperlipidemia seem to have a lower prevalence among highlanders, thus indicating a reduced risk of developing atherosclerosis and stroke (Faeh et al., 2009; Aryal et al., 2017; Ortiz-Prado et al., 2019).

Long-term exposure to hypobaric hypoxia seems to be linked to healthier blood lipid profiles when compared with those of residents living at sea level (Mohanna et al., 2006; Siqués et al., 2007; Vats et al., 2013). According to the report by Gonzales et al., who studied 158 people living at 4,100 m, the fraction of non-high-density lipoprotein (HDL) cholesterol and triglycerides is directly associated with the value of hemoglobin, and their increase, in turn, is associated with higher diastolic blood pressure. More specifically, high hemoglobin levels were directly associated with higher levels of total cholesterol, low-density lipoprotein (LDL), HDL, and triglycerides, and no association was found between hemoglobin and glucose (Gonzales and Tapia, 2013). Al Riyami et al. (2015) showed that altitude was the most significant factor affecting HDL-C, followed by gender, serum triglycerides, and finally the 2-h post prandial plasma glucose. Also, Vats et al. (2013) pointed out that in the process of acclimatization to high altitude, there is an increase in the diastolic blood pressure and heart rate, in addition to an increase in HDL levels. Although these responses have been described previously, the differences between the two indigenous groups, which shared the same ancestry but adapted to life at very different altitudes, have never been reported before. This fortunate circumstance gives us a great opportunity to understand the role of exposure to the altitude as a causal determinant of these differences disregarding genetic ancestry.

The objective of the current report is to explore the plasma lipid profile and cardiovascular risk differences among autochthonous Kiwcha populations permanently living at low and high altitudes.

## Methodology

### Study Design

A cross-sectional analysis of the differences in plasmatic lipid profiles and cardiovascular risk was carried out in two populations of Kiwcha natives from Ecuador living at two different elevations.

### Setting

This study was carried out in Ecuador in two geographically different areas, the Andes mountain range and the Amazon Basin.

### Participants

This study was carried out in 134 women and 79 men who voluntarily accepted to participate in the study. All the participants who voluntarily agreed are members of the Kiwcha indigenous group from Ecuador. The high-altitude group came from Oyacachi, a small Kiwcha community located at 3,800 m of elevation while the low-altitude group was the Kiwcha people living at Limoncocha, located at 230 m of elevation.

### Inclusion Criteria

The study was conducted among healthy volunteers of both sexes without any type of comorbidity or chronic disease, between the ages of 18 and 85, who were born and currently residing in Oyacachi (high-altitude group), and in Limoncocha (low-altitude group).

### Exclusion Criteria

Volunteers who were under 18 years of age, those who were born in another community, and those who do not habitually reside in the aforementioned parishes were excluded from the study.

### Variables and Outcomes

Sociodemographic variables, such as age, sex, marital status, and place of residence were recorded. Vital signs were obtained by our team that included five doctors in the field. To assess arterial blood pressure, we used an upper arm blood pressure monitor 3 Series® Model: BP7100 from OMRON based on the American Heart Association (AHA) Recommendations for Blood Pressure Measurement (Smith, 2005). To evaluate body fat percentage, body mass index (BMI), and body weight we use the Omron Body Composition Monitor & Scale HBF-514C manufactured by OMRON which measures fat using the bioimpedance method. The temperature was measured using a portable Non-Contact Professional Medical Grade Infrared Thermometer. For the entire blood laboratory work, we included the following lipid profile serum parameters: LDL (mg/dl), HDL (mg/dl), triglycerides (mg/dl), and total cholesterol (mg/dl). We have also included mean fasting blood glucose levels (mg/dl) and clinical parameters including systolic and diastolic blood pressure, heart and respiratory rate, height and body weight, and BMI. We computed the 10-year risk of heart disease or stroke for ages between 40 and 79 years using the AHA risk calculator (http://Kiwcha.cvriskcalculator.com/). A blood sample was used to extract RNA to determine ancestry roots from both populations and confirm that they share the same genetic traits.

### Outcome

The main outcome is to determine the different lipid profiles and cardiovascular risk ratios among genotype-matched Kiwcha indigenous people who live at high altitude vs. their counterparts who live at low altitude.

### Data Sources

Individual-level sociodemographic information, place of residence, and past medical history were obtained *in situ* in both communities. A complete physical examination including measurement of body weight and height, arterial blood pressure, body temperature, resting heart, and respiratory rate, and arterial oxygen saturation was performed.

### Study Size and Sample Size Calculation

In terms of the number of patients required to achieve significance, the sample size (*n*) and margin of error (*E*) were given by the following formula:


$$x=Z({}^{c}/{\;}_{100}{)}^{2}r(100-r)$$

$$n={\;}^{N_{x}}/{\;}_{\left[\left(\text{N}-1\right){\text{E}}^{2}+\text{x}\right]}$$

$$E=Sqrt{[}^{(\text{N}-\text{n})\text{x}}{/}_{\text{n}(\text{N}-1)}]$$

where *N* is the population size (*n* = 570 in Oyacachi and *n* = 890 in Limoncocha), (*r*) is the fraction of expected responses (50%), and *Z*(*c*/100) is the critical value for the confidence level (*c*). The total number of medical and physical evaluations required to achieve statistical significance was 82 for the high-altitude group and 96 for the low-altitude group. Through a nonprobability convenience-based sampling technique, 118 patients (40 men and 78 women) were included for Limoncocha and 95 patients were included for (39 men and 56 women) for Oyacachi.

### Data Analysis

Descriptive statistics were used to analyze and visualize differences between the two populations. A chi-square test was performed to check the association or independence of categorical variables. When the expected values were <5 in any of the categories, Fisher's exact test or Spearman's test were used when the variable had evident asymmetries with histograms prior to the selection of the test. Additionally, a two-way ANOVA test was performed to determine the influence of gender and altitude of the populations on the continuous dependent variables, followed by age correction.

To compare the population ancestries for Oyachachi and Limoncocha, a *t*-test was performed, considering individual genotypes. Normal distribution and equal variance were assumed; the test concludes (*p* = 0.05) that there is no difference between any of the continental contributions of the three founding ethnic groups considered.

All statistical analyses accepted significance when *p*-value <0.05. Calculations were completed using the IBM Corp. Released 2014. IBM SPSS Statistics for Windows, Version 24.0. Armonk, NY: and R Core Team software 2018 version 3.5.1. Cartography was generated using QGIS Development Team 2.8 and all the references were managed using the open-source software Zotero 5.0.85.

### DNA Extraction and Analysis of Ancestry Ratios

To compare the ancestry of the two populations, a subsample of 47 unrelated individuals (30 Oyacachi vs 17 Limoncocha) was selected. We looked for a subsample among all the individuals to identify those subjects who did not have any first-order degree of consanguinity, a condition that is based on our laboratory protocol for ancestry analysis. DNA extraction was performed from FTA cards (GE Healthcare) by the Chelex method. The extracts were then diluted to a concentration of 5 ng/ul using the NanoDrop 2000 UV-Vis spectrophotometer (Thermo Scientific, Waltham, MA; Walsh et al., 1991). 46-plex autosomal ancestry informative deletion-insertion markers (46-plex AIMs-InDel) were amplified. Fluorescent amplicons were sized by capillary electrophoresis in Pop-7 polymer using a genetic analyzer ABI 3130 (Applied Biosystems, Austin, TX). Alleles were named by the software Genemapper V 3.1 (Life Technologies, Carlsbad, CA) following nomenclature described by Pereira et al. (2012). Taking into account trihybrid historic mixture in Ecuador (Santangelo et al., 2017; Toscanini et al., 2018; Zambrano et al., 2019), inference of ancestry proportions was obtained considering the admixture model with *K* = 3 (based on runs consisting of 100,000 burn-in steps, followed by 100,000 Markov Chain Monte Carlo (MCMC) using the STRUCTURE V2.3.4 software (Pritchard et al., 2000).

All runs were made without any prior information on the origin of samples and only considered the genetic background for the ancestral continental populations based on reference samples: European, EUR (*n* = 158); African, AFR (*n* = 105); and Native American, NAM (*n* = 64). Reference genotypes were extracted from the diversity panel of the Human Genome Diversity Project-Center d'Etude du Polymorphisme Humain (HGDP-CEPH). The populations selected as comparative groups for Africa were: Angola (*n* = 1), Botswana (*n* = 4), Central African Republic (*n* = 23), Congo (*n* = 13), Kenya *(n* = 11), Lesotho (*n* = 1), Namibia (*n* = 6), Nigeria (*n* = 22), Senegal (*n* = 22), and South Africa (*n* = 2); for South America: Brazil (*n* = 22), Colombia (*n* = 7), and Mexico (*n* = 35); and for Europe: France (*n* = 52), Italy (*n* = 49), Orkney Islands (*n* = 15), and Russia (*n* = 42).

### Ethical Consideration

Full ethical approval was obtained (#MED.EOP.17.01) throughout the Universidad de las Americas bioethics committee (CEISH). All patients voluntarily signed informed consent. For people who could not read or write, an official community translator and a family member capable of understanding what was described in the document were used to explain the entire context of the project and ensure that there were no doubts about it. To protect the identity and autonomy of patients, all personal information was coded to ensure anonymity.

## Results

### Demographic Results

A total of 213 subjects were recruited in both communities. 52.9% (*n* = 118) were included from the Limoncocha low altitude group and 47.1% (*n* = 95) from the Oyacachi high altitude group. In general, women represented 63% (*n* = 134) of the entire cohort and men 37% (*n* = 79).

### Age and Sex Differences

In the low altitude group, 66% were women (*n* = 78) and 34% (*n* = 40) were men, whereas at high altitude, 59% (*n* = 56) were women and 41% (*n* = 41%) were men (Table 1).

**Table 1:** Demographic characteristics, weight, height, and body mass index (BMI) of the two populations in relation to sex.

		**Low altitude (230 m)**	**High altitude (3800 m)**	**(%) Diff**.	***p*-value**
Median age (IQR)	Men	42.0 (30.0–52.0)	36.0 (25.0–57.0)	15.4	0.137
	Women	41.0 (30.0–59.0)	36.0 (29.0–48.0)	13	
Young adult (18–35 Kiwcha)	Men	24 (54.5)	27 (67.5)	11.8	0.475
	Women	45 (57.0)	41 (73.2)	9.3	0.086
Adult (36–64 Kiwcha)	Men	15 (34.1)	10 (25.0)	40	0.475
	Women	19 (24.1)	11 (19.6)	53.3	0.086
Elderly (>65 Kiwcha)	Men	5 (11.4)	3 (7.5)	50	0.475
	Women	15 (19.0)	4 (7.1)	115.8	0.086
Weight (kg)	Men	74.2 ± 10.8	60.3 ± 8.71	20.7	**0.0001**
	Women	62.7 ± 14.4	60.8 ± 8.3	3.1	
Height (cm)	Men	159.9 ± 6.3	155.5 ± 9.93	2.8	**0.001**
	Women	149.2 ± 7.0	152.6 ± 8.6	2.3	
Normal weight (18.5–24.9)	Men	5 (12.5)	21 (53.8)	123.1	**0.001**
	Women	25 (32.1)	20 (35.7)	22.2	**0.036**
Overweight (25–29.9)	Men	22 (55.0)	16 (41.0)	31.6	**0.001**
	Women	31 (39.7)	29 (51.8)	6.67	**0.036**
Obesity type I and II (30–39.9)	Men	7 (17.5)	2 (5.1)	111.1	**0.001**
	Women	12 (15.4)	7 (12.5)	52.6	**0.036**
Extreme obesity (>40)	Men	6 (15.0)	0 (0.0)		**0.001**
	Women	10 (12.8)	0 (0.0)		**0.036**

*Mean ± SD; IQR, interquartile range. Bold values are Statistically significant difference at 95% confidence level*.

The median age for the low altitude group was 41 years and 36 years for men and women, respectively. The sex–age intergroup differences were not significant for all the groups (Table 1).

### Weight and BMI Differences

In relation to weight, we found that women at low altitudes are on average 1.9 kilos lighter than women at high altitudes (60.84 kg ± 8.33 kg), but this difference was not statistically significant (*p* = 0.374). Men living at high altitudes are 20.7% lighter than their counterparts at low altitudes (*p* < 0.0001). We did not find any underweight adult subjects in any group; however, we found the proportion of overweight patients and those with obesity type I, II, and extreme obesity to be significantly higher among low altitude dwellers (Table 1).

### Vital Signs Differences by Sex and Elevation

We found that arterial blood pressure tends to be higher in men (106/75 mmHg) than in women (102/70 mmHg). Nevertheless, this small difference is not significant. The Mean Arterial Blood Pressure (MAP) and systolic blood pressure were 6.2 and 7.5%, respectively, lower in men from the high-altitude group when compared to men from the low altitude group. These differences are statistically significant (*p* = 0.01 and 0.029) (Table 2).

**Table 2:** Description of the main vital signs of both populations including arterial blood pressure, heart and respiratory rate, temperature, and blood peripherical oxygen saturation.

**Vital sign**		**Low altitude (230 m)**	**High altitude (3,800 m)**	**(%) Diff**	**Sig**.
SBP	Women	100 (90–110)	104 (90–120)	3.9	0.029
	Men	110 (100–120)	102(99–110)	7.5	
DBP	Women	70.0 (60–80)	70 (70–80)	0	0.016
	Men	80 (70–80)	70 (60–80)	13.3	
MAP	Women	74.9 ± 8.7	77 ± 11	3	0.010
	Men	80.2 ± 8.3	75.3 ± 8.6	6.2	
HR' (m ±SD)	Women	73. ± 10	66.0 ± 11	10.1	0.911
	Men	71 ± 13	65 ± 9	8.8	
RR' (m ±SD)	Women	18 ± 18–19	18 ± 18–23	0	0.346
	Men	18 ± 18–22	18 ± 18–21	0	
SpO_2_%	Women	98 ± 97–99	93 ± 91–94	5.2	0.076
	Men	98 ± 97–98	93 ± 92–94	5.2	
T°	Women	36.7 ± 36.3–37.2	36 ± 36–36	0.8	0.565
	Men	36.5 ± 36.3–37.2	36.1 ± 35.7–36.7	1.1	

*SBP, Systolic blood pressure; DBP, Diastolic blood pressure; HR', Heart Rate Beats per minute; RR', Respiratory rate breaths per minute; m ±SD, Mean ± standard deviation; IQR, interquartile range; SpO_2_%, peripheral Blood Oxygen saturation; T°, Body Temperature; MAP, Mean Arterial Blood Pressure (mmHg)*.

In terms of heart rate frequency (beats per minute), high altitude dwellers have a 9.4% lower heart rate; nevertheless, gender and level of altitude did not influence heart rate calculated by a two-way ANOVA and the difference was not statistically significant (*p* = 0.911).

**Figure 1:**
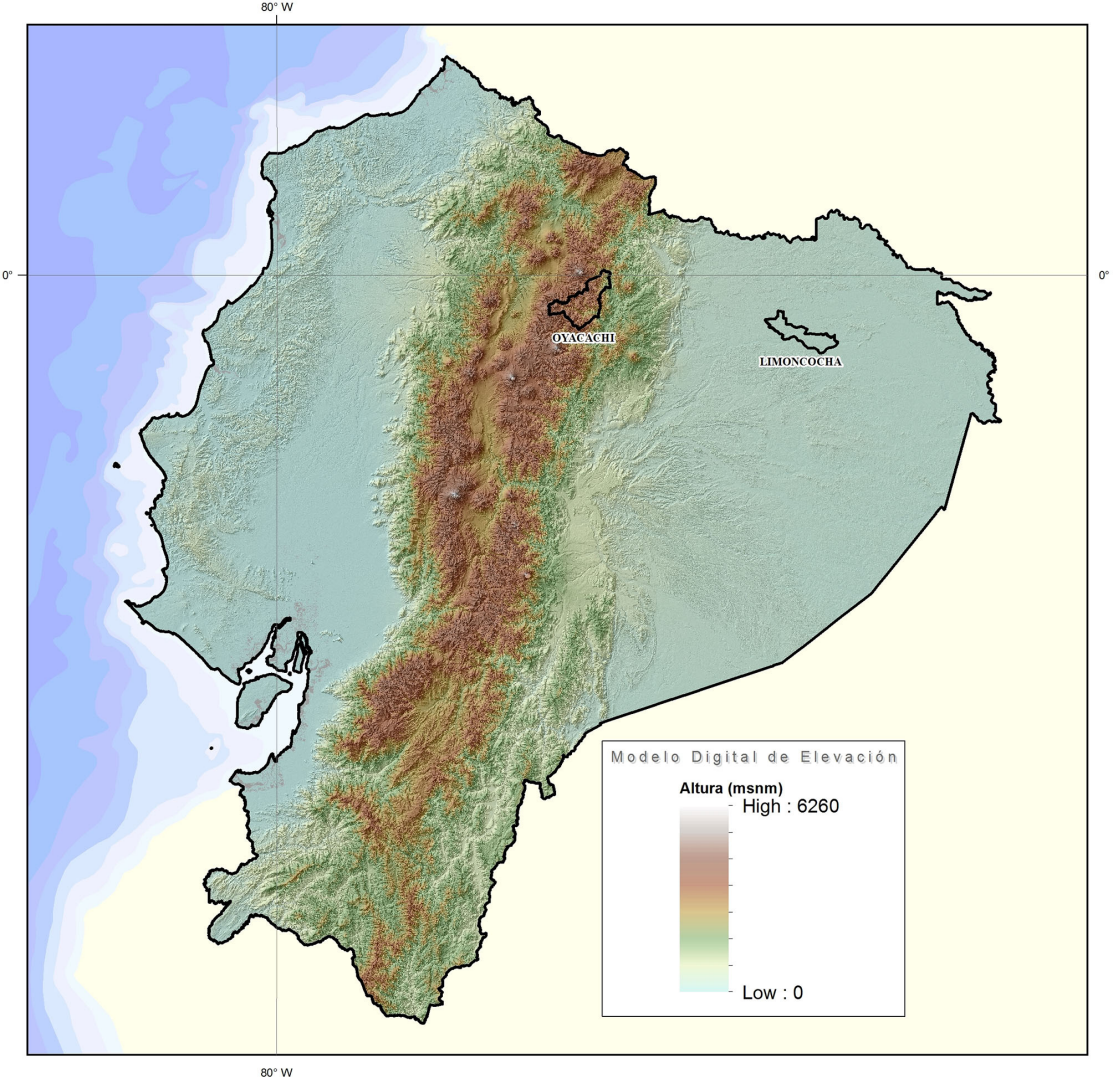
Vital signs differences according to sex and altitude.

Despite this, we found a 5.2% lower peripheral blood oxygen saturation for the low altitude group. Gender and altitude did not influence SpO_2_% calculated by a two-way ANOVA (*p* = 0.076) (Table 2 and Figure 1).

### Complete Blood Count (CBC), Biochemical Analysis, and Cardiovascular Risk Analysis Between Groups

**Figure 2:**
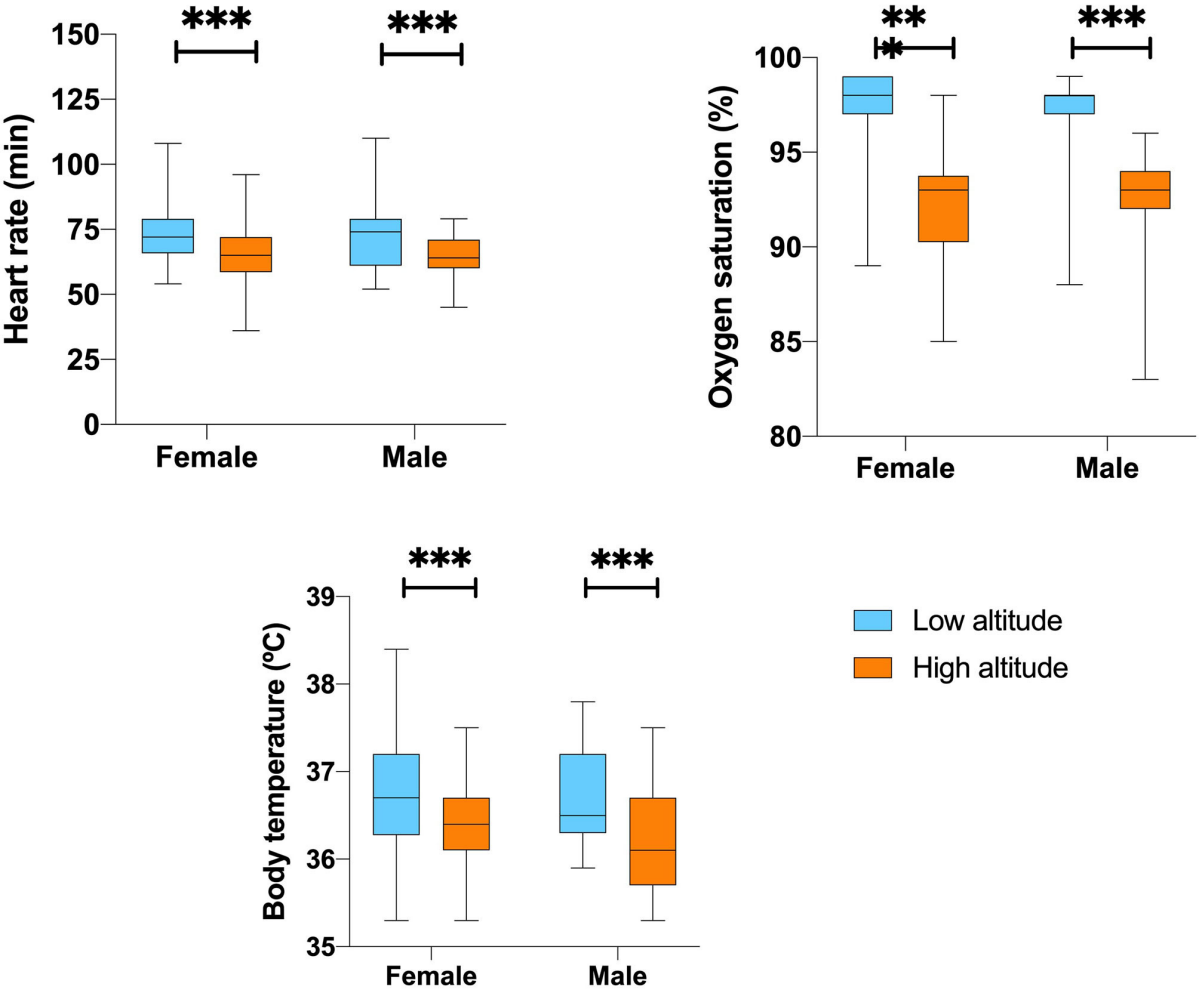
The differences of Hematological parameter according to sex by altitude. **,***Represents outliers values.

Differences in white blood cell counts were not observed among the low and high-altitude groups (Table 3). For red blood cells (RBCs) count and microscopic features, we found that high altitude dwellers have higher cells counts and high levels of hematocrit and hemoglobin; however, they have smaller RBCs that contain less hemoglobin per erythrocyte. Nevertheless, after correcting for age, altitude, and sex, the differences did not reach the 5% established significant level (Figure 2).

**Table 3:** Complete blood count (CBC) and blood biochemical analysis in low and high-altitude dwellers.

	**Women**		**Men**		
	**Low altitude (230 m)**	**High altitude (3,800 m)**	**Low altitude (230 m)**	**High altitude (3,800 m)**	***P*-value**
Lymphocytes	7.0 ± 2.0	6.0 ± 2.0	7.0 ± 2.0	6.0 ± 1.0	0.163
Neutrophiles	56.0 ± 8.0	55.0 ± 9.0	55.0 ± 7.0	52.0 ± 9.0	0.416
Lymphocytes	36.0 (32.0–42.0)	36.0 (31.0–40.0)	36.0 (32.0–40.0)	37.0 (32.0–46.0)	0.763
Monocytes	6.0 (5.0–7.0)	6.0 (5.0–7.0)	7.0 (6.0–9.0)	7.0 (6.0–8.0)	0.418
Eosinophiles	2.0 (1.0–2.0)	1.0 (1.0–3.0)	2.0 (2.0–3.0)	2.0 (1.0–3.0)	0.190
Hematocrit	41.0 (38.0–42.0)	47.0 (45.0–49.0)	45.0 (43.0–47.0)	52.0 (50.0–54.0)	0.515
Hemoglobin	13.45 ± 1.01	15.23 ± 1.10	15.31 ± 1.11	17.06 ± 1.01	0.897
RBC	4.0 ± 4.0–5.0	5.0 ± 5.0–5.0	5.6 ± 5.3–6.0	6.0 ± 5.0–6.5	0.363
Platelets	263.00 ± 52.00	276.00 ± 47.00	257.00 ± 53.00	257.00 ± 55.00	0.368
MCV	94.00 ± 4.00	92.00 ± 4.00	93.00 ± 3.00	91.00 ± 4.00	0.826
MCH	32.0 (30.0–32.0)	30.0 (29.0–31.0)	32.0 (31.0–32.0)	30.0 (29.0–31.0)	0.250
MCHC	33.0 (33.0–34.0)	33.0 (32.0–33.0)	34.0 (34.0–35.0)	33.0 (33.0–33.0)	**0.031**
Glucose	89 (83–95)	89 (84–92)	91.0 (83.0–97.0)	90.0 (84.0–95.0)	0.411
Cholesterol	174 ± 37	193.0 ± 28.0	167.0 ± 38.0	196.0 ±30.0	0.275
Triglycerides	127 (90–179)	90 (73–143)	133.0 (106.−180)	110.0 (78.0–146.0)	0.438
HDL	46.0 (40–55)	56.0 (46.0–71.0)	41.0 (38.0–47.0)	49.0 (44.0–60.0)	0.610
LDL	98.0 ± 32.0	113.0 ± 22.0	93.0 ± 34.0	117.0 ± 26.0	0.278
AHA Heart Risk	2.0 (1.0–5.0)	1.0 (1.0–5.0)	3.0 (2.0–5.0)	5.0 (2.0–9.0)	0.461

*RBC, Red Blood Cells; MCV, Mean corpuscular volume; MCH, Mean corpuscular hemoglobin; MCHC, Mean corpuscular hemoglobin concentration. Bold value is statistically significant difference at 95% confidence level*.

**Figure 3:**
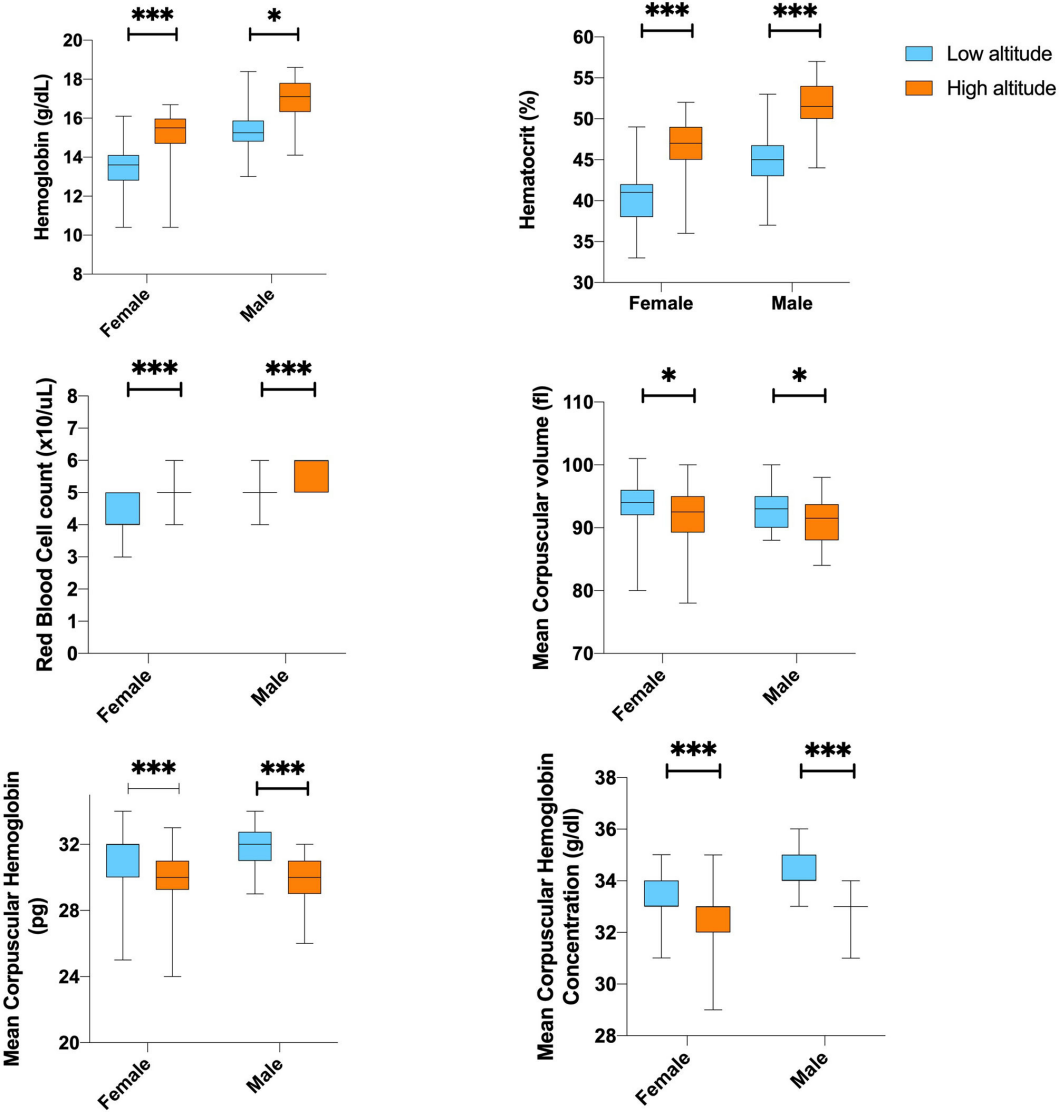
Blood biochemistry and lipid profile difference value according to sex by altitude. **,***Represents outliers values.

In terms of serological biochemical parameters, we did not find significant differences in mean fasting blood glucose levels or lipid profiles. For instance, low altitude dwellers have significantly lower total cholesterol, lower HDL, and lower LDL values for both sexes. Nevertheless, triglycerides are on average 26% lower among high altitude dwellers. Despite these clinical differences, after correcting for age, sex and altitude, they did not reach significance at 5% level (Figure 3).

When computing a 10-year risk of heart disease or stroke using the atherosclerotic cardiovascular disease (ASCVD) algorithm published in the 2013 American college of cardiology (ACC)/AHA Guideline on the Assessment of Cardiovascular Risk, we did not find any statistically significant difference between groups (Table 3).

Gender and the level of altitude did not influence the overall blood biochemical analysis calculated by the two-way ANOVA. However, the combination of gender and altitude showed to significantly affect mean corpuscular hemoglobin concentration (*p* = 0.031), even when this relationship considers adjustment for age (*p* = 0.033).

**Figure 4:**
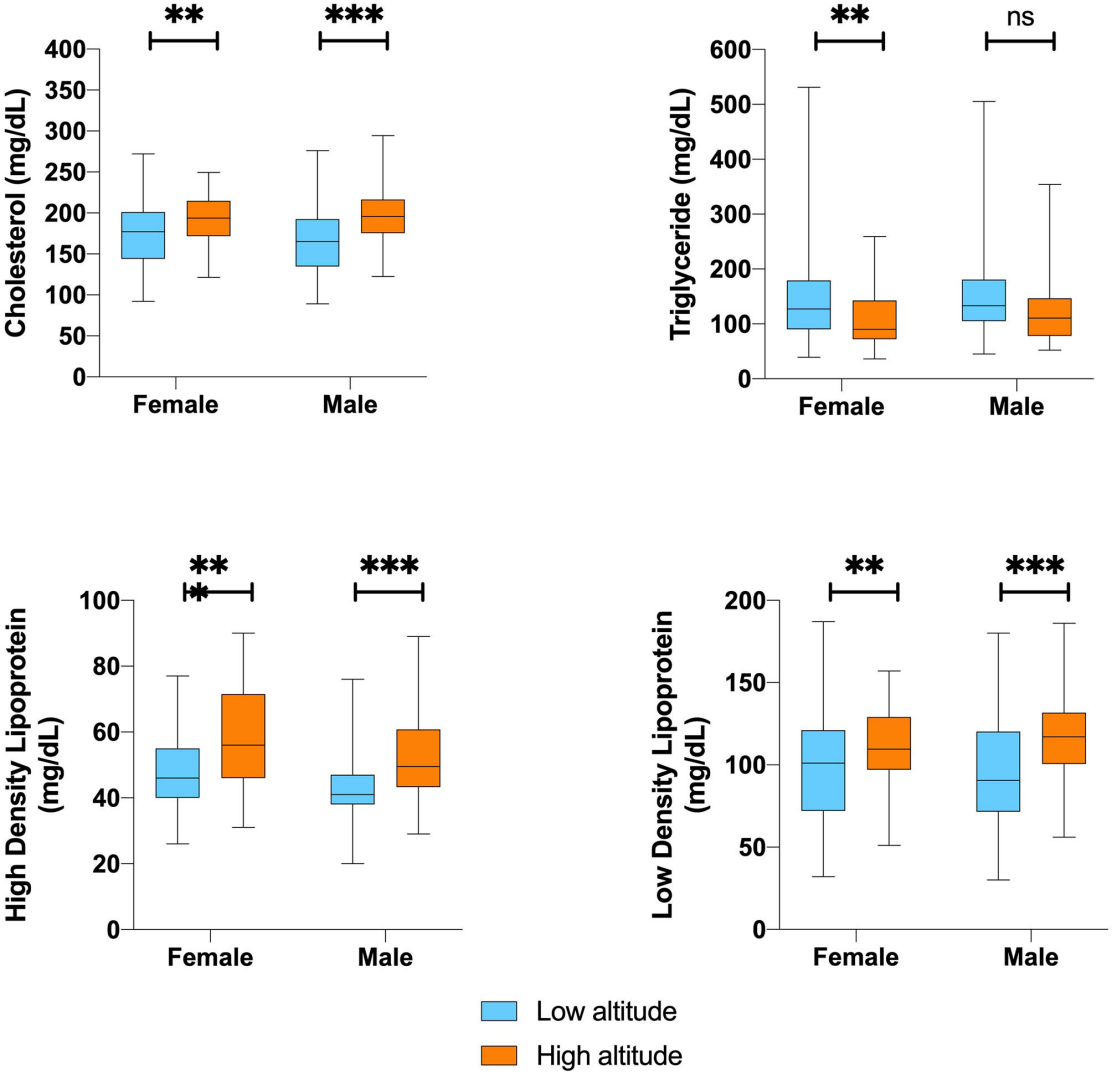
Ancestry and main significant clinical and laboratory findings differences between low and high-altitude group. **,***Represents outliers values.

Ancestry proportions between Oyacachi and Limoncocha are not statistically different. Both communities retain Native American ancestry >97% and vary slightly in the European (0.6 vs. 1.4%) and Afro (0.3 vs. 0.7%) contributions. However, they are highly conserved populations in general, Oyacachi being the most mixed, considering the data obtained from this sample and analysis (Figure 4).

## Discussion

Our study demonstrates that hematological, biochemical, and some clinical parameters differ between the two populations that share the same ancestral origin but have resided for centuries at two different geographical locations. Most of them are not influenced by gender or elevation. While all these differences have been described in different populations, this is the first time that we have been able to determine them as genetically controlled populations that share genetic, sociodemographic, and economical similarities, and geographically distinct territories (GAD Oyacachi, 2019; GAG Limoncocha, 2019).

Some of the differences that we have found, especially anthropometric distinctions are probably due to the adaptive processes. These processes have been described in several investigations that have explained how humans chronically exposed to high altitudes become more fit to function under hypoxic conditions (Julian and Moore, 2019).

The results of our study compare anthropometric differences in a genotype-controlled indigenous adult population living at low (230 m) and high altitudes (3,800 m). When analyzing the data, we observe that in general, women from high altitudes are slightly lighter and slightly taller than women from the lowlands (Merrill, 2020); nevertheless, men from high altitudes are significantly shorter and lighter than men from low altitudes. Our findings are similar to those reported in Bolivia by Leatherman et al. (1984). This study conducted an anthropometric survey among 138 men from rural mountainous areas of Bolivia (3,700 m) and concluded that men from high altitudes are shorter and lighter than their low altitude counterparts (Leatherman et al., 1984). Among Quechuas, a similar native group from Peru, Toselli et al. (2001) found individuals shorter at high altitudes in relationship to their corporal mass (Toselli et al., 2001). In contrast to earlier findings, however, no evidence of these results was detected by Khalid (1995) when they showed that high altitude residents from Saudi Arabia were significantly heavier and taller than the low altitude control group. These differences between two populations (the Andean and the Saudis) could demonstrate differences in terms of adaptation, something that has been described extensively before (Moore et al., 1998, 2011; Beall, 2007; Tyagi et al., 2008; Moore, 2017a).

On the other hand, women from high altitudes have a higher proportion of obesity than their low altitude counterparts, possibly due to cultural conditions that force women to stay at home cooking while men leave their houses to work (Khalid, 2007; Lin et al., 2018).

It has been hypothesized that at least 5% of high-altitude natives from Peru possess a newly discovered gene named *FBN1*. This gene seems to be associated with favoring high altitude Andean natives with low stature and possibly thicker skin (Pennisi, 2018). It is well-known that high altitude dwellers and animals are often smaller, an evolutionary response to the shortage of food or oxygen and thicker skin, which may help shield the body from intense UV radiation in such places (West, 2012; Pennisi, 2018).

It is well-known that weight among newborns is significantly lower among high-altitude neonates than their sea-level counterparts (Al-Shehri et al., 2005; Hoke and Leatherman, 2019), a situation that might continue not only during pregnancy but during the 1st years of childhood and adolescence (Lichty et al., 1957; Iannotti et al., 2009; Moore et al., 2011).

The fact that newborns are smaller has to do with an adaptive process that aims to reduce oxygen consumption by the fetus, being more efficient to deliver oxygen to a smaller organism through a smaller placenta (Krüger and Arias-Stella, 1970; Zamudio, 2003; Dolma et al., 2021).

Besides anthropometric differences, high altitude residents had superior lung capacities, enhanced vascularity, a blunted ventilatory response to sustained hypoxia and lower exercise ventilation, and overall superior efficiency of O_2_ transport, utilization, and distribution (Zhuang et al., 1993; Brutsaert et al., 2005; Moore, 2017a; Ortiz-Prado et al., 2019). In the present study, we tried to identify whether there are physiological differences that are not necessarily due to sociocultural, social, economic, or differences in habit. According to the latest data from local governments in both Oyacachi and Limoncocha, the schooling rate, mortality rate, economic dependence, and access to health care services are similar in both parishes (GAD Oyacachi, 2019; GAG Limoncocha, 2019). Both parishes have only one health center provided regulated by the Ministry of Public Health (MoH).

In relation to high altitude lifestyle differences, habits, and endogenous preconditioning, gathering data is a complex task. Different populations have different eating habits, different lifestyles, and they usually subsist in a way different than their low-land counterparts (Westerterp, 2001; Lundby et al., 2006; Li and Zhao, 2015; Brutsaert, 2016). The data about risk factors available in Ecuador suggest that people living in provinces from the highlands consume more alcohol (17.1 vs. 9.1%) and smoke more (6.5 vs. 2.5%) than the people living at lower altitudes (Freire et al., 2015). In a general nationwide analysis, the National Institute of Statistics and Censuses of Ecuador (INEC) reported that people in the coast region seem to have a higher consumption of carbohydrates (36 vs. 30%) than those living in the highlands (INEC, 2018). Although these data on dietary variability could be extrapolated to the population living at high altitudes in Ecuador, it is well-known that people visiting high altitude locations have a significant loss in appetite and an accelerated metabolism that might speed up weight loss (Karl et al., 2018; Rausch et al., 2018).

The aforementioned similarities and some differences shared by both populations might not be fully responsible for our findings. We believe that physiological, hematological, and lipid profile differences have a genetic, respiratory, circulatory, and adaptive origin although most of them were not influenced by gender or elevation. For instance, we found that heart rate (HR') within the high-altitude population was 7 beats per minute slower than those at low altitudes and men always report lower MAP than women. This may be explained by the significantly high polycythemia described at high altitudes (Winslow, 1984). The higher the number of RBCs, the easier the oxygen transport, translating into a reduced cardiac output among adapted populations (West et al., 2007; Miggitsch et al., 2009). In a recently published analysis, Holmström et al., suggested that a lower metabolic rate and greater parasympathetic activity might be common among highlanders (Holmström et al., 2020).

Having smaller RBCs, higher hemoglobin concentrations, lower MAP, and other differences might be, in part, attributed to their adaptational process experienced for centuries of living at different altitudes (Moore et al., 2011; Moore, 2017b). The Kiwcha population living at 230 m above sea level migrated further south centuries ago, while the Kiwcha group living above 3,800 m above sea level found a place to successfully thrive at a high altitude (Cardoso et al., 2012). When comparing the data obtained from both indigenous groups located at low and high altitudes, we did not find differences in the profile of their white blood cells; however, the size of RBCs and hemoglobin composition were found to be clinically different as expected and noted in several previous studies (Beall et al., 1998; Beall, 2006; Storz, 2007).

The difference in the number of RBCs and their size is expected since the low availability of oxygen at high altitudes due to the low barometric pressure causes a positive response on erythropoiesis and the subsequent production of RBCs (Zhong et al., 2015; Akunov et al., 2018; Ortiz-Prado et al., 2019).

**Figure 5:**
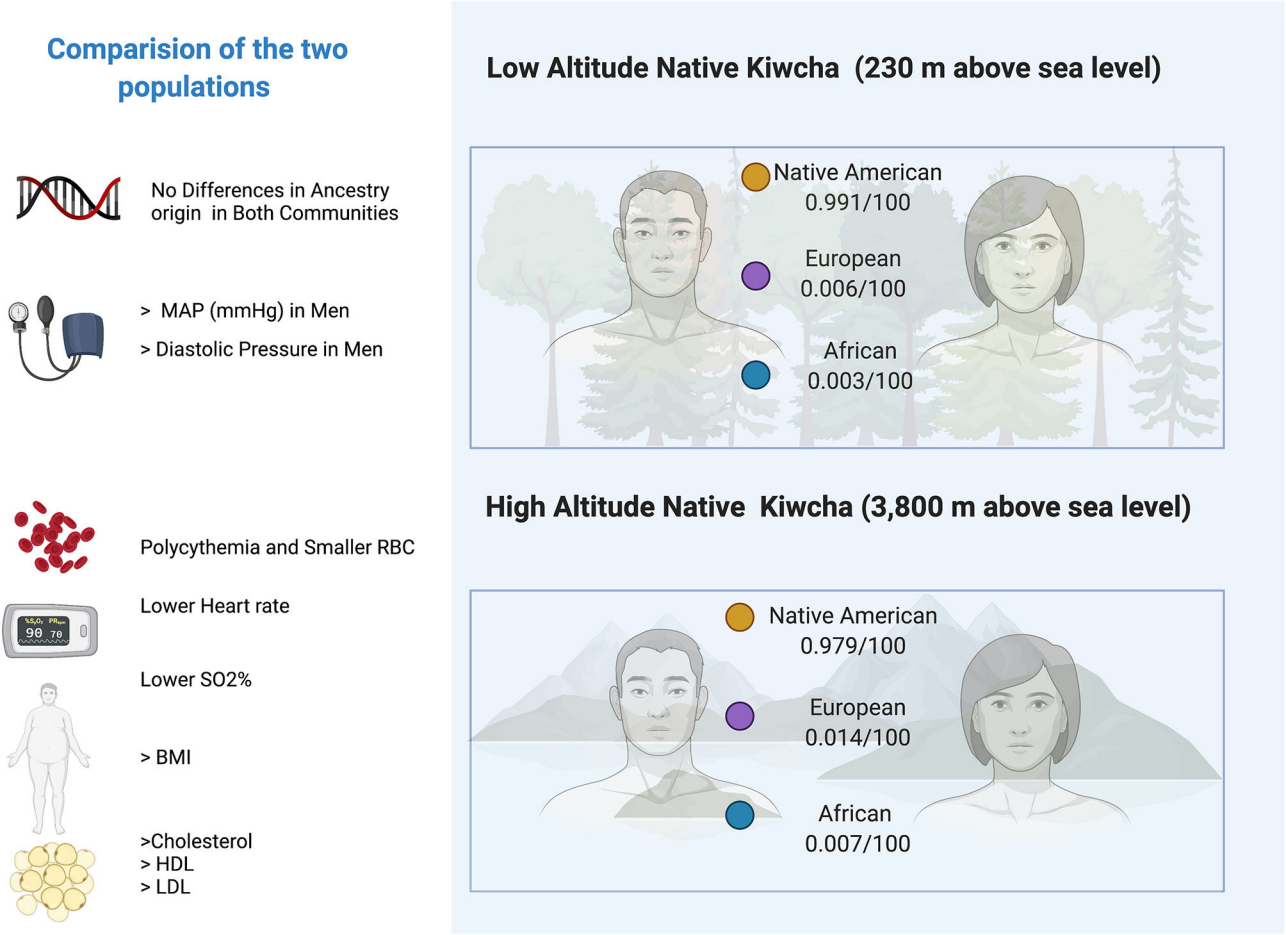
Digital infographic showing the main clinical differences between the inhabitants of Limoncocha (low altitude) and Oyacachi (high altitude).

The observed differences within the high-altitude population (Figure 5) might indicate an increased oxygen-carrying capacity (Samaja et al., 2003). The higher the production of the RBCs the thicker the blood, therefore, adaptative mechanisms based on a slightly reduced size of the RBCs and lower hemoglobin concentration within the erythrocyte might confer an evolutive advantage, reducing the risk of blood stasis (Stobdan et al., 2017).

Also noticeable are the differences in plasma lipid profile, as the group located at low altitude is more prone to having higher levels of triglycerides, especially among women, whereas the group located at a high altitude presents higher total cholesterol serum level and LDL and HDL levels which differ from men to women. Partially supporting these findings, high rates of hypercholesterolemia have been described in adult populations above 3,600 m that inhabit Peru and Tibet (Mohanna et al., 2006; Sherpa et al., 2011). However, the study of the influence of altitude on the lipid profile parameters has not been able to show causality due to the wide variability in the available data. A study by Ranhotra and Sharma, when comparing two populations of indigenous Khasis adults living at high and low altitudes, showed a decrease in total cholesterol and LDL of high-altitude residents, accompanied by a decrease in triglyceride levels at high altitudes (Ranhotra and Sharma, 2010). Similarly, a study by Siqués et al. carried out in natives of low altitudes who were exposed for 8 months at a height of 3,550 m did not reveal changes in total cholesterol levels and was accompanied by an increase in the concentration of triglycerides after altitude exposure (Siqués et al., 2007). Therefore, the influence of external factors such as physical activity, sedentary lifestyle, diet, and tobacco consumption has a considerable impact on the lipid profile of the altitude inhabitants.

These differences in habit patterns, lipid profile, and even in the ratio between obese and non-obese populations could be associated with lower mortality caused by cerebrovascular and cardiovascular diseases at high altitudes (Faeh et al., 2009; Burtscher et al., 2021).

Faeh et al. (2009) and Burtscher et al. (2021), provided data supporting the statement that living at a moderate altitude (1,000–2,000 m) elicits beneficial effects on all-cause mortality for both sexes, including diseases of the circulatory system in Switzerland and Austria, respectively (Faeh et al., 2009; Burtscher et al., 2021).

In the Kiwcha's case, the geographical isolation, and consequent sociodemographic and cultural factors that have been exposed over time, determine some behavioral differences between the Kiwcha inhabits of Oyacachi and Limoncocha living at different altitudes, which also may have an influence on our findings (GAD Oyacachi, 2019; GAG Limoncocha, 2019).

Despite not having found significant differences in the risk for the development of heart disease and stroke, lower rates of coronary heart disease and stroke have been observed in the European population living at moderate altitudes (Faeh et al., 2009; Burtscher, 2016), and a progressive decrease in mortality from coronary heart disease and stroke has been observed as the altitude increases. External factors such as hypoxia level and solar radiation can also play a role. However, these effects are mainly observed at moderate elevations (around 2,000 m) in contrast to higher elevations (above 3,000 m; Moore, 2001; Faeh et al., 2009; Burtscher, 2016).

The study of these Andean populations confers an interesting opportunity to explore differences in a well-controlled group. The Oyacachi Kiwcha population (high altitude) and the Limoncocha group have evolved differently thanks to their geographical differences. In our context, having two populations that are genetically similar but have adapted to their landscapes for more than 500 years may provide important information on the mechanisms that could be linked to adaptation. As the adaptation to chronic hypoxia is polygenic, molecular adaptations may differ from those found in other parts of the planet, as has been seen among people living in the Himalayas or the mountainous areas of Ethiopia (Moore, 2001; Azad et al., 2017).

For instance, a recently published study suggests that both genetic predisposition and environmental exposure determine the size and function of human organs such as the spleen (Holmström et al., 2020). Although this information has not been compared with Andean natives, the increased spleen size found among Sherpas might also be linked to an improved circulating hemoglobin function (Holmström et al., 2020).

In an extensive literature review by Azad et al. (2017), the authors described the genomic implications of the adaptation of different organisms to high altitude (Azad et al., 2017). They described how a series of genetic components gave rise to the different bio-molecular pathways that regulate oxygen transport, the circulatory system functioning or the overall erythrocyte, oxygen, and hemoglobin homeostasis (Azad et al., 2017).

We suggest that several molecular and physiological mechanisms that have yet to be revealed might play a direct role in explaining some of the differences described in this study. Although numerous factors and variables could not be controlled, the reported findings provide new insights about an understudied population.

## Limitations

The main limitation of this study was the absence of a dietary and exercise assessment, as diet massively alters blood lipid profile. Another limitation was that despite obtaining a significant sample size to carry out this study, not the entire population belonging to these indigenous communities that met the inclusion criteria was willing to participate. So, even if it is a small probability, it cannot rule out that the inclusion of the data corresponding to those people who did not participate could produce variations in our results or even alter our interpretation. Another potential weakness is the gender asymmetry in the sample size because men were a lower number of participants than women.

## Conclusion

Permanent life at both altitudes induced well-known adaptive responses in Kiwcha dwellers: increased number of erythrocytes, hemoglobin concentration, hematocrit level, and serum glucose level. Although we have found remarkable differences in the plasma lipid profile between the populations at the two altitudes, these alterations did not seem to be influenced by altitude, sex, or age.

## Data Availability Statement

The original contributions presented in the study are included in the article/supplementary material, further inquiries can be directed to the corresponding author/s.

## Ethics Statement

The studies involving human participants were reviewed and approved by Universidad de las Americas, CEISH. The patients/participants provided their written informed consent to participate in this study.

## Author Contributions

EO-P was fully responsible for the conceptualization, data collection, and elaboration of the study. EO-P, DP, and JM-M participated in drafting the manuscript equally and are fully responsible for it. DP, JM-M, and DD visited indigenous communities and collected samples. KS-R, JI-C, and EO-P contributed to the data collection and the construction of figures and tables. EO-P, MC, EV, JI-C, and GV contributed to the descriptive statistical analysis and the discussion section of the manuscript. GB was responsible for DNA extraction and analysis of ancestry ratios. EO-P critically reviewed the entire manuscript and produced several comments prior to submission. All the authors have read and approved the final version of the manuscript.

## Funding

This project was fully funded by Universidad de las Americas, Quito, Ecuador during the internal annual call for projects scheme.

## Conflict of Interest

The authors declare that the research was conducted in the absence of any commercial or financial relationships that could be construed as a potential conflict of interest.

## Publisher's Note

All claims expressed in this article are solely those of the authors and do not necessarily represent those of their affiliated organizations, or those of the publisher, the editors and the reviewers. Any product that may be evaluated in this article, or claim that may be made by its manufacturer, is not guaranteed or endorsed by the publisher.

## Abbreviations

LDL: Low-density lipoprotein
HDL: High-density lipoprotein
AHA: American Heart Association
HGDP-CEPH: Human Genome Diversity Project - Centre d'Étude du Polymorphisme Humain
EUR: European
AFR: African
NAM: Native American
AIMs: Ancestry Informative Markers
IQR: Interquartile Range
DS: Deviation Standard.
